# Stemness Correlates Inversely with MHC Class I Expression in Pediatric Small Round Blue Cell Tumors

**DOI:** 10.3390/cancers14153584

**Published:** 2022-07-22

**Authors:** Linda Müller, Maik Kschischo, Christian Vokuhl, David Stahl, Ines Gütgemann

**Affiliations:** 1 Department of Mathematics and Technology, University of Applied Sciences Koblenz, RheinAhrCampus, 53424 Remagen, Germany; lmuelle2@hs-koblenz.de (L.M.); kschisch@hs-koblenz.de (M.K.); 2 Institute of Pathology, University Hospital Bonn, 53127 Bonn, Germany; christian.vokuhl@ukbonn.de; 3 Department I of Internal Medicine, University Hospital of Cologne, 50937 Cologne, Germany; david.stahl1@uk-koeln.de

**Keywords:** stemness, MHC class I, pediatric tumors, small round blue cell tumors

## Abstract

**Simple Summary:**

Tumors occurring at a young age are distinct from tumors in older individuals, clinically and pathologically. As small round blue cell tumors (SRBCTs), they often show a resemblance to stem cells and immature precursor cells during embryonal development. Recently, immunotherapy has become an option for a subset of patients with limited success. We observed that in almost all the pediatric SRBCT types investigated (*n* = 1134) there was an inverse relationship, when comparing genes highly expressed in stem cells with genes encoding MHC class I molecules. MHC class I molecules are important in tumor cell recognition by cytotoxic T cells. We suspect that these tumors are derived from multipotent precursor cells that naturally show a low MHC class I expression and a lack of immune recognition necessary for prenatal proliferation and development.

**Abstract:**

Recently, immunotherapeutic approaches have become a feasible option for a subset of pediatric cancer patients. Low MHC class I expression hampers the use of immunotherapies relying on antigen presentation. A well-established stemness score (mRNAsi) was determined using the bulk transcriptomes of 1134 pediatric small round blue cell tumors. Interestingly, MHC class I gene expression (HLA-A/-B/-C) was correlated negatively with mRNAsi throughout all diagnostic entities: neuroblastomas (NB) (*n* = 88, *r* = −0.41, *p* < 0.001), the Ewing’s sarcoma family of tumors (ESFT) (*n* = 117, *r* = −0.46, *p* < 0.001), rhabdomyosarcomas (RMS) (*n* = 158, *r* = −0.5, *p* < 0.001), Wilms tumors (WT) (*n* = 224, *r* = −0.39, *p* < 0.001), and central nervous system-primitive neuroectodermal tumors CNS-PNET (*r* = −0.49, *p* < 0.001), with the exception of medulloblastoma (MB) (*n* = 76, *r* = −0.24, *p* = 0.06). The negative correlation of MHC class I and mRNAsi was independent of clinical features in NB, RMS, and WT. In NB and WT, increased MHC class I was correlated negatively with tumor stage. RMS patients with a high expression of MHC class I and abundant CD8 T cells showed a prolonged overall survival (*n* = 148, *p* = 0.004). Possibly, low MHC class I expression and stemness in pediatric tumors are remnants of prenatal tumorigenesis from multipotent precursor cells. Further studies are needed to assess the usefulness of stemness and MHC class I as predictive markers.

## 1. Introduction

While cancer is among the leading causes of death among children and adolescents, in terms of total numbers, cancer is a rare event during childhood and young adulthood. Immunotherapies have entered and revolutionized the field of cancer therapy, showing survival benefits in patients with tumors at advanced stages. Recently, these immunomodulatory approaches have also become feasible options for selected subsets of pediatric cancer patients [1].

Histologic diagnosis and tumorigenesis differ between pediatric/young adult patients and older individuals. Tumorigenesis in adult patients frequently occurs because of an accumulation of mutations over time and a progression from hyperplasia, dysplasia, and in situ tumors to invasive tumors is seen. In pediatric cancer patients, the tumorigenesis, as well as the histological appearance, often takes the form of small round blue cell tumors (SRBCTs), mimicking early progenitor cells. Genetically, fewer somatic mutations but a higher prevalence of germline alterations in cancer predisposition genes are observed [2].

Examples of tumors that are thought to arise prenatally are medulloblastoma (MB), neuroblastoma (NB), Ewing’s sarcoma family of tumors (ESFT), central nervous system-primitive neuroectodermal tumors (CNS-PNET), rhabdomyosarcoma (RMS), and Wilms tumor (WT). Cytomorphologically, these tumors consist of small and immature-looking discohesive cells with a high nuclear-to-cytoplasmic ratio, thus called “small round blue” cells. These tumors are thought to arise prenatally from immature progenitor cells during embryonic or fetal development [3].

Tumors are frequently described as being immunologically hot or cold with a presumed implication for the effectiveness of particular immunotherapeutic approaches. Childhood brain tumors are known to be relatively immunologically cold due to the paucity of mutations [4]. The major histocompatibility complex (MHC) is a multi-gene family encoding a series of cell surface proteins that function to bind and present antigens to the adaptive immune system. MHC class I is expressed by all nucleated cells and, together with the beta-2-microglobulin chain, plays a crucial role in cancer immunosurveillance resulting in peptide MHC class I recognition on tumor cells by cytotoxic T cells. It is also a prerequisite for efficient immunotherapy, especially using immune-checkpoint inhibitors [1]. One of the most common mechanisms by which tumors evade the host immune response is by the downregulation of MHC class I molecule expression, thereby rendering endogenous or therapeutic anti-tumor T cell responses ineffective [5]. Several mechanisms of MHC class I downregulation have been identified, yielding different phenotypes [6]. Dysregulated NF-κB causes both a low MHC class I expression and the decreased expression of genes involved in the antigen-presenting machinery which is required for the appropriate conformational folding of the MHC class I molecule. Few studies have examined MHC class I expression in pediatric cancers in a limited number of patients [5], showing a low expression of MHC class I in high-risk NB patients [7] and negativity in poorly-differentiated RMS [5]. The majority of ESFTs exhibit a partial absence of MHC class I expression, with lung metastases consistently being negative [8]. MHC class I expression in MB is associated with a poor prognosis, a pattern opposite of most other cancers [9].

The reason why low MHC class I expression in pediatric tumors is so common is unclear. It hampers a productive cytotoxic endogenous immune response against neoantigens and consequently also limits the response to immunotherapeutic approaches. Immunotherapy for pediatric solid tumors remains in the early stages of development, and significant clinical benefit has yet to be realized, with anti-disialoganglioside 2 (GD2) for neuroblastoma representing a notable exception [10,11].

We previously evaluated stemness features and the composition of the tumor immune microenvironment in pediatric tumors [12]. Tumor progression involves the loss of differentiation and the acquisition of stem-cell-like features [13,14]. Here, we focused on the analysis of MHC class I expression in pediatric tumors in order to see whether antigen-presenting molecule expression in bulk transcriptomes depends on stemness proximity as defined by gene expression using an mRNA stemness index, the mRNAsi [13]. The mRNAsi was developed as a predictive model for stemness using known embryonal and pluripotent stem cell datasets [13]. In adult type cancers, a high mRNAsi is correlated with a dedifferentiated oncogenic state and is generally higher in metastases [13]. In pediatric tumors, mRNAsi is correlated with histology (WT), stage, molecular subtype, and survival (NB) [12]. Interestingly, here we identified a strong negative correlation between the mRNAsi and MHC class I expression of pediatric SRBCTs.

## 2. Materials and Methods

### 2.1. Gene Expression Data

In this study, publicly available gene expression microarray data from 1134 pediatric SRBCTs across six malignancies (ESFT, RMS, WT, CNS-PNET, NB, MB) were analyzed. The gene expression profiles (GEPs) per dataset (series accession, GSE) and clinical–pathological data were retrieved from the Gene Expression Omnibus (GEO) [15,16] and its corresponding publications. Analysis was confined to two microarray platforms to avoid cross-platform bias: Affymetrix HG-U133A (GEO accession number GPL96) and Affymetrix HG-U133 Plus 2.0 (GEO accession number GPL570). Table 1 summarizes the GSE datasets used in this study. Mean MHC class I gene expression was defined as the mean of HLA-A, HLA-B, and HLA-C gene expression. The mean with 95% confidence interval (95% CI) gene expression values are provided in Supplementary Table S1. Analyses were always performed within a GSE study. Different GSE studies were not pooled and were not analyzed together.

### 2.2. Microarray Preprocessing

The Affymetrix array raw data were downloaded as CEL files from the GEO [17] and the probes were aggregated to HUGO gene symbols. All microarray studies were normalized according to the “robust multi-array average” (RMA) [18] method prior to the analysis using the “affy” package in Bioconductor and R (R Foundation for Statistical Computing, Vienna, Austria).

### 2.3. Calculation of a Gene Expression-Based Stemness Index (mRNAsi)

The gene expression-based stemness index mRNAsi was calculated for all samples within this cohort of pediatric SRBCTs [13]. This stemness index is a predictive model using a one class logistic regression (OCLR) algorithm on pluripotent stem cell samples from the Progenitor Cell Biology Consortium (PCBC) dataset [19,20] to train the stemness signature. The OCLR model enables the identification of specific cell types in heterogeneous cell populations [21]. The mRNA expression-based signature contains a gene expression profile including 12,892 genes. To set the stemness score, the Spearman correlation between the weighted genes and the corresponding gene expression values is calculated. The workflow to generate the mRNAsi is available on https://bioinformaticsfmrp.github.io/PanCanStem_Web/ (accessed on 30 September 2019). We applied the stemness index model to score all samples within the cohort. RMA- and log2-transformed normalized values of the gene expression data were used to generate the mRNAsi. The stemness index was subsequently mapped to the (0,1) range.

### 2.4. Assessment of Tumor-Infiltrating T Cells by CIBERSORT

We used CIBERSORT to examine the relative fractions of tumor-infiltrating T cells, using the LM22 signature matrix with 1000 permutations (other parameters were left at default values) as previously described [22]. The leucocyte signature comparison matrix used within the CIBERSORT algorithm was validated on both microarray platforms used in this study, Affymetrix HG-U133A (GEO accession number GPL96) and Affymetrix HG-U133 Plus 2.0 (GEO accession number GPL570) [22].

### 2.5. Statistical Analysis

Statistical analysis was performed using the software package IBM SPSS (Chicago, IL, USA), Statistics for Windows (version 28), and R (R Foundation for Statistical Computing, version 4.1.2). Mean value comparisons were performed with the Mann–Whitney-U test, two-sample *t*-test, or Kruskal–Wallis test as indicated in the figure and table legends, depending on the number of compared groups and the data distribution. Variances were tested for equality using Levene’s test for homogeneity of variances. If the variances were heterogenous, the Welch approximation to the degrees of freedom was used. To examine the effect size of differences between the compared means, the effect size was calculated according to Cohen. Correlation analyses were performed according to the Spearman’s rank correlation coefficient method (R: “*cor.test()*”). The Benjamini–Hochberg (BH) procedure [23] was used to correct for multiple testing errors with a false discovery rate of 0.05 (R: “*p.adjust()*”). Where it has not been otherwise specified in the figure legends, data are presented as box plots with horizontal bars representing the median. Survival analysis was performed using the Kaplan–Meier method and the log-rank test. The optimal cut-off point defining high and low MHC class I expression and CD8 T cell count was set at the point with the most significant (log-rank test) separation using the web-based tool “cutoff Finder” [24]. *p*-values of less than 0.05 were considered statistically significant.

## 3. Results

### 3.1. MHC Class I and Non-Classical MHC Expression in Pediatric SRBCTs

We systematically analyzed the gene expression of MHC class I (HLA-A/-B/-C) and non-classical MHC (HLA-E/-F/-G) genes in 1134 pediatric SRBCTs across six malignancies (CNS-PNET, ESFT, MB, NB, RMS, and WT). HLA-A followed by HLA-B and HLA-C showed the highest gene expression values across all of the included malignancies (Supplementary Table S1). The gene expression levels of non-classical MHC genes such as HLA-E and HLA-F were moderate throughout all malignancies. The MHC class I expression values within the largest cohorts of all six malignancies (CNS-PNET: GSE73038, *n* = 59; ESFT: GSE34620, *n* = 117; MB: GSE37418, *n* = 76; NB: GSE16476, *n* = 88; RMS: GSE92689, *n* = 158; WT: GSE31403, *n* = 224) are shown in Supplementary Table S1. Genes of the antigen-processing machinery and regulation (IRF1, IRF2, NF-κB1, NF-κB2, NLRC5, TAP1, TAP2, TAPBP) were not significantly expressed throughout all of the analyzed tumor transcriptomes (Supplementary Table S1).

### 3.2. Negative Correlation of MHC Class I and Stemness (mRNAsi)

**Figure 1:**
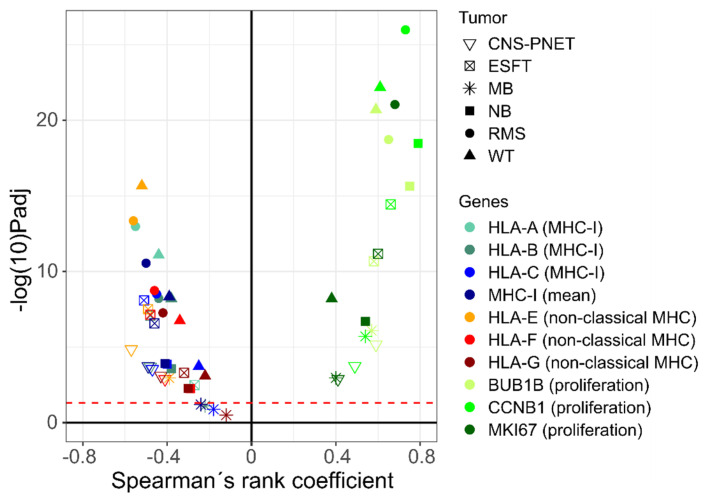
Mean MHC class I expression correlates negatively with stemness (mRNAsi) in CNS-PNET (*n* = 59, *r* = −0.49, *p* < 0.001), in ESFT (*n* = 117, *r* = −0.46, *p* < 0.001), in MB (*n* = 76, *r* = −0.24, *p* = 0.06), in NB (*n* = 88, *r* = −0.41, *p* < 0.001), in RB (*n* = 158, *r* = −0.5, *p* < 0.001), and in WT (*n* = 224, *r* = −0.39, *p* < 0.001). Non classical MHC class I proteins (HLA-E/-F/-G) were also negatively correlated with the mRNAsi, while proliferation genes such as BUB1B, CCNB1, and MKI67 were positively correlated with the mRNAsi (similar findings were observed for other previously reported proliferation genes [25]: BUB1, CCNB1, CCNE1, MYBL2, PLK1; data not shown). The x-axis shows the Spearman’s rank coefficient *r* and the y-axis shows the -log(10)P(adj). Abbreviations: Ewing’s sarcoma family of tumors, ESFT; medulloblastoma, MB; neuroblastoma, NB; central nervous system-primitive neuroectodermal tumor, CNS-PNET; rhabdomyosarcoma, RMS; Wilms tumor, WT.

**Figure 2:**
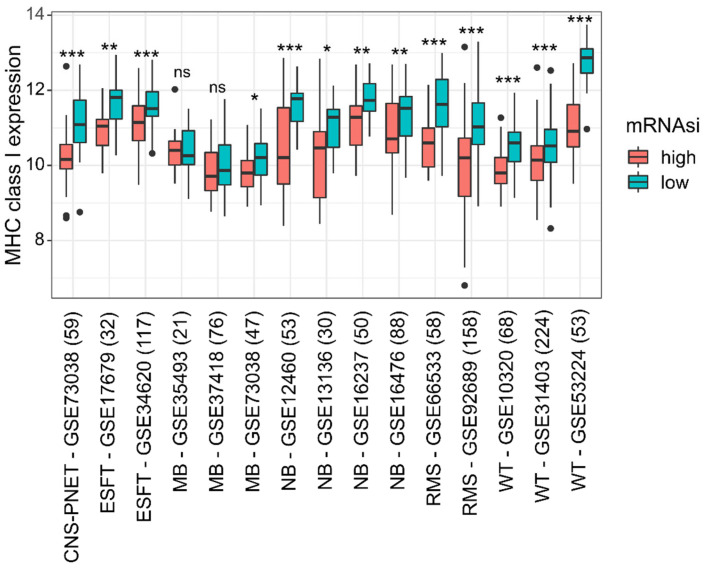
Low mRNAsi correlates with higher MHC class I gene expression levels in test and validation cohorts of pediatric small round blue cell tumors. Each GSE study was divided into a mRNAsi low (cyan) and mRNAsi high (red) group according to the median. The numbers of samples are given in parentheses. Two-sample *t*-test, * *p* < 0.05, ** *p* < 0.01, *** *p* < 0.001; not significant, ns. Abbreviations: Ewing’s sarcoma family of tumors, ESFT; medulloblastoma, MB; neuroblastoma, NB; central nervous system-primitive neuroectodermal tumor, CNS-PNET; rhabdomyosarcoma, RMS; Wilms tumor, WT.

**Figure 3:**
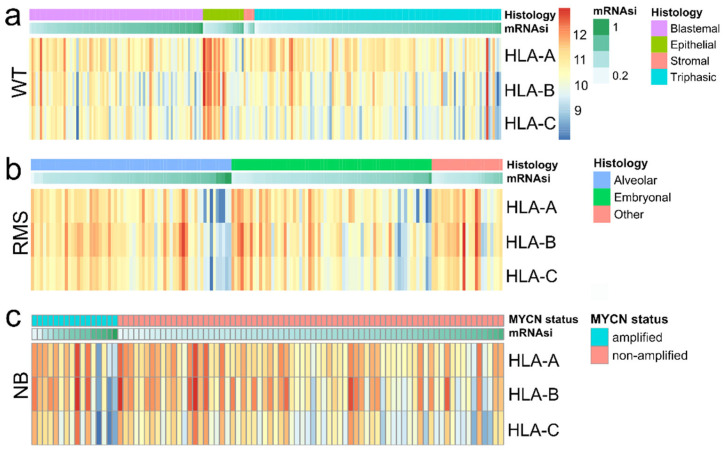
Tumors with a high stemness index (mRNAsi) are associated with low HLA-A/-B/-C expression independent of clinical–pathological data such as histological subtype and MYCN status. Gene-expression heatmaps of HLA-A, HLA-B, and HLA-C stratified for mRNAsi and clinical–pathological data in (**a**) WT (GSE31403, *n* = 224), (**b**) RMS (GSE92689, *n* = 158), and (**c**) NB (GSE16476, *n* = 88). Cohorts were first subdivided into histological and molecular subtype as shown, and subsequently sorted according to the mRNAsi level ranging between 0 and 1 (see Methods). Significant observations (Spearman’s rank correlation coefficient) are shown as follows—WT (GSE31403, *n* = 224): HLA-A (*r* = -0.44, *p* < 0.001), HLA-B (*r* = −0.38, *p* < 0.001), HLA-C (*r* = −0.25, *p* < 0.001); RMS (GSE92689, *n* = 158): HLA-A (*r* =−0.55, *p* < 0.001), HLA-B (*r* = −0.44, *p* < 0.001), HLA-C (*r* = −0.45, *p* < 0.001); NB (GSE16476, *n* = 88): HLA-A (*r* = −0.41, *p* < 0.001), HLA-B (*r* = −0.38, *p* < 0.001), HLA-C (*r* = −0.4, *p* < 0.001). Results were validated within further cohorts as shown in Figure 2 and Supplementary Table S2. Abbreviations: neuroblastoma, NB; rhabdomyosarcoma, RMS; Wilms tumor, WT.

A negative correlation between MHC class I expression and the stemness index mRNAsi was found in all pediatric SRBCTs except those of MB (Figure 1, Supplementary Table S2). The highest correlation coefficient values between MHC I (mean of HLA-A/-B/-C) and mRNAsi were found in RMS (*r* = −0.50, *p* < 0.001, GSE92689) and the lowest values in WT (*r* = −0.39, *p* < 0.001, GSE31403). The correlations between MHC class I and mRNAsi within the remaining malignancies were as follows: NB (*r* = −0.41, *p* < 0.001, GSE16476), ESFT (*r* = −0.46, *p* < 0.001, GSE34620), CNS-PNET (*r* = −0.49, *p* < 0.001, GSE73038), and MB (*r* = −0.24, *p* = 0.06, GSE37418). These findings were validated in separate datasets (Figure 2, Supplementary Table S2). Importantly, in NB, RMS, and WT, the correlation between mRNAsi and MHC class I expression was independent of MYCN status and histological subtype; it was observed, e.g., in both alveolar RMS (*r* = −0.45, *p* < 0.001, *n* = 65, GSE92689) and embryonal RMS (*r* = −0.61, *p* < 0.001, *n* = 65, GSE92689), in blastemal WT (*r* = −0.4, *p* < 0.001, *n* = 81, GSE31403), epithelial WT (*r* = −0.81, *p* < 0.001, *n* = 19, GSE31403), and triphasic WT (*r* = −0.29, *p* = 0.002, *n* = 115, GSE31403), and also in MYCN-amplified NB (*r* = −0.63, *p* = 0.009, *n* = 16, GSE16476) and non-MYCN-amplified NB (*r* = −0.39, *p* = 0.001, *n* = 72, GSE16476) (Figure 3, Supplementary Table S2). Interestingly, those non-classical MHC class I genes with moderate to high expression such as HLA-E and HLA-F also showed a negative correlation with mRNAsi across all malignancies (Figure 1). In WT with predominantly epithelial histology and in WT with a low mRNAsi, the highest expression levels of the HLA-A/-B/-C genes were observed (GSE31403) (Figure 3).

### 3.3. Proliferation and Its Positive Correlation with Stemness (mRNAsi)

Next, the gene expression of individual proliferation genes was assessed and compared with the stemness index mRNAsi. The proliferation genes BUB1B, CCNB1, and MKI67 were correlated positively with stemness (Figure 1). Strongest correlation was found in NB (*r* = 0.79, *p* < 0.001, GSE16476, gene: *CCNB1*) and the lowest positive correlation values in WT (*r* = 0.38, *p* < 0.001, GSE31403, gene: *MKI67*) (Figure 1).

### 3.4. MHC Class I Expression Correlates with Clinical–Pathological Features and Survival

**Figure 4:**
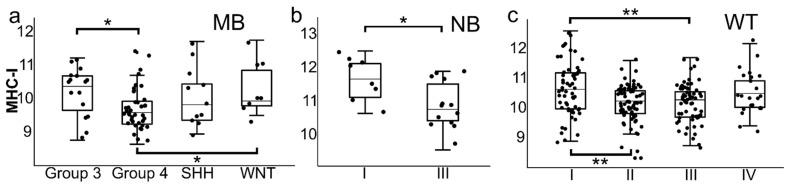
(**a**) MHC class I expression was significantly lower in Group 4 medulloblastomas compared to Group 3 and WNT (*p* = 0.027, Kruskal–Wallis test) medulloblastomas. (**b**) Stage I neuroblastomas showed a higher MHC class I expression compared with stage III neuroblastomas (*p* = 0.025, Mann–Whitney-U test). (**c**) In Wilms tumors, MHC class I expression was significantly higher in stage I tumors compared to stage II and stage III (*p* = 0.004, Kruskal–Wallis test) tumors. * *p* < 0.05, ** *p* < 0.01. The four principal molecular subgroups of medulloblastoma WNT, SHH, Group 3, and Group 4 are based on distinct transcriptional and epigenetic signatures defining clinically relevant patient subsets [26]. WNT and SHH medulloblastomas are primarily driven by activation of the wingless/int-1 (WNT) and sonic hedgehog (SHH) signaling pathways, while Group 3 and Group 4 medulloblastomas are molecularly and clinically heterogeneous. Abbreviations: medulloblastoma, MB; neuroblastoma, NB; Wilms tumor, WT.

**Figure 5:**
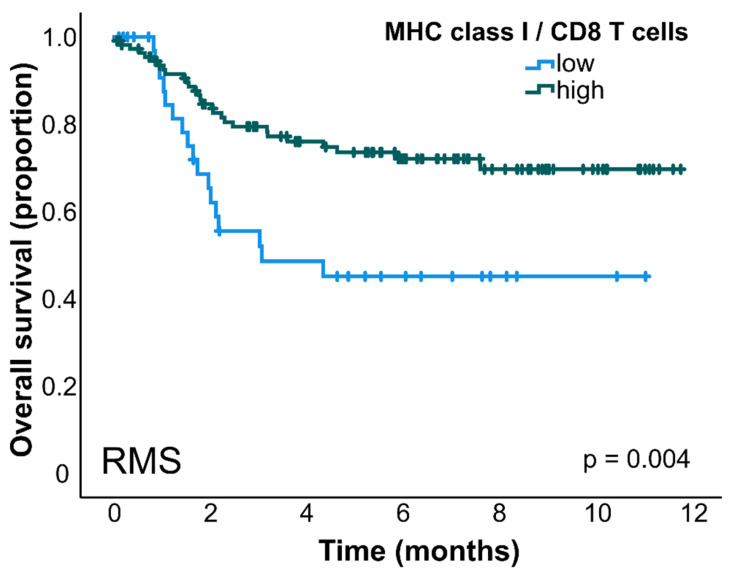
Rhabdomyosarcoma patients with a high MHC class I expression and abundant CD8 T cell infiltration showed a prolonged overall survival (*p* = 0.004, log-rank test, GSE92689, *n* = 148). CIBERSORT was used to examine the relative fraction of tumor-infiltrating CD8 T cells. MHC class I/CD8 T cells high was defined as a high expression of MHC class I and high infiltration of CD 8 T cells; MHC class I/CD8 T cells low was defined as a low expression of MHC class I and/or low infiltration of CD8 T cells (see Methods, [24]). Abbreviation: rhabdomyosarcoma, RMS.

MHC class I gene expression correlated with the clinical–pathological data in this cohort of pediatric SRBCTs. In NB and WT, the highest MHC class I expression was found in stage I tumors compared with higher tumor stages (Figure 4). Group 4 MBs showed a lower MHC class I expression compared to Group 3 MBs and WNT MBs (Figure 4). There was no correlation seen between MHC class I expression and other clinical–pathological features such as histological subtype or MYCN status. The survival analysis revealed that RMS patients with a high MHC class I expression and a high abundancy of intratumoral CD8 T cells (CIBERSORT) had a longer overall survival (*p* = 0.004, Figure 5).

## 4. Discussion

This in silico analysis of gene expression data from 1134 pediatric SRBCTs across six malignancies revealed a strictly negative correlation of MHC class I expression and stemness for which a well-established stemness score (mRNAsi) was used as a surrogate marker. This correlation was seen throughout all diagnostic tumor entities except those of MB, independent of clinical–pathological characteristics (Figure 1, Figure 2 and Figure 3; Supplementary Table S2). Importantly, this negative correlation was found in different molecular and histological subtypes, i.e., in embryonal and alveolar RMS, MYCN-amplified and non-amplified NB, and WT of different histologic subtypes (Figure 1 and Figure 3, Supplementary Table S2). Positive and negative correlations of MHC class I and stemness have been observed in adult tumor types; however, these studies present heterogenous results [8,16,17]. 

MHC class I molecules present endogenous peptides to CD8 T cells, and tumor cells evolve multiple strategies to avoid elimination by CD8 T cells [27]. MHC class I downmodulation is observed in adult epithelial cancers, resulting in the avoidance of anti-tumor immunity and a poor response to immunotherapy [28]. Cancers can also evade immune elimination by expressing non-classical MHC class I molecules (MHC Ib), such as HLA-E and HLA-G. Notably, non-classical MHC molecules also correlated negatively with mRNAsi (Figure 1).

In contrast to adult tumor types that show the loss or downmodulation of MHC class I sporadically due to various mechanisms, our results suggest that pediatric tumors inherently show a negative correlation of MHC class I and stemness, most likely as a remnant of embryonal development.

The expression of most components of the MHC class I antigen-presentation pathway, including MHC class I heavy chains, beta-2-microglobulin, immunoproteasome subunits, TAP, Tapasin, and ERAP1, are coordinately regulated by similar gene control elements in their promotors and/or enhancers [27], including the NLRC5-enhanceosome, NF-κB, and IRF1/IRF2. MHC class I molecules were frequently expressed in this cohort of pediatric SRBCTs at different levels, whereas the expression of genes involved in antigen promotion and regulation such as Tapasin, TAP1, TAP2, ERP57, and TAPBP were generally low (Supplementary Table S1).

Multiple mechanisms have been elucidated on MHC class I downregulation [28] including the structural loss/downmodulation of regulatory factors or epigenetic modifications. In NB, low MHC class I expression is due to posttranslational mechanisms that, interestingly, can be retrieved by NF-κB stabilization therapeutically through retinoids—known to induce differentiation, apoptosis, and the inhibition of tumor cell proliferation [29].

It has been well established that cancers employ multiple mechanisms to evade the immune system, including the downmodulation of MHC class I, rendering them less susceptible to immunotherapy [30]. During embryonal development, MHC class I expression is tightly regulated during the gestational process, first on rare stromal cells and at later timepoints on epithelial cells [31], possibly reflecting the need for proliferating precursor cells during development to avoid immune recognition and rejection. In pediatric tumors, stemness and low MHC class I expression may be part of the epigenetic and genetic regulatory network from immature progenitor cells during prenatal development, which is different from adult tumorigenesis in which MHC class I downmodulation is an active acquired mechanism to avoid immune recognition.

Our findings are in line with earlier results showing low MHC class I expression in MB and CNS-PNET [32,33,34] and in NB [35].

Interestingly, a comparison of pediatric SRBCTs showed no correlative values for mRNAsi and MHC class I in MB (Figure 1 and Figure 2, Supplementary Table S2). MHC class I expression in MBs may be a reflection of the MHC class I expression status of precursor cells in the external granular layer [32]. The WHO classification currently includes four histologically (classic, desmoplastic/nodular, MB with extensive nodular, and large cell/anaplastic MB) and four genetically defined groups (WNT-activated; SHH-activated, either TP53 mutated or TP53 wild type; and non-WNT/non-SHH of either Group 3 MB or Group 4 MB) [33]. MHC class I expression in our analysis was lower in Group 4 medulloblastomas compared to Group 3 and WNT tumors (Figure 4). Interestingly, cerebellar prenatal external granular cells that later migrate into the postnatal layers express MHC class I molecules, whereas they are negative in other cells within the developing cerebellum by immunohistochemistry [32]. One possibility is that the heterogeneity of MHC class I expression in various molecular subgroups in MB may be a remnant of the genetic program in different cerebellar lineage precursor cells [34]. In contrast to other tumor types, MHC class I expression is associated with a poor prognosis in some MBs [9], possibly because of ERK activation by incomplete so-called conformer MHC class I molecule expression, deficient of beta-2-microglobulin and/or peptide expression [32].

A positive correlation was observed between proliferation and stemness. It remains to be seen whether proliferation rates defined by antibody staining in situ correlate with stemness by gene expression in these tumors, and/or whether a combination of KI67-positive nuclei and MHC class I expression could be used as a surrogate predictive marker for immune-mediated regression or response to immunotherapy.

Interestingly, NBs sometimes show spontaneous regression [36], associated with differentiation into benign ganglioneuroma. It would be interesting to investigate in such cases whether differentiation is associated with MHC class I upregulation and respective changes in the immune microenvironment of such tumors. In WT, very early observations saw parallels between a lack of MHC class I expression by immunohistochemistry, while differentiated tubular structures were MHC class I-positive, paralleling renal development with MHC class I expression in glomeruli from 8 weeks and in tubules from 13 weeks gestational age onwards [37].

MHC class I expression in combination with a high infiltration of CD8 T cells was associated with an improved overall survival in RMS (Figure 5). This is an indicator that, in RMS also, differentiating tumors contain more adaptive immune cells, allowing better immune surveillance by the endogenous adaptive immune response, as well as a better response to immunotherapies.

The fact that molecular/histological subtypes and stage within a given tumor correlates with MHC class I expression (Figure 4) and mRNAsi [12] supports the theory that MHC class I expression and stemness are features recapitulated in tumors derived from these cells. We speculate that maturation of pediatric SRBCTs may lead to MHC class I upregulation and increased immune recognition by the endogenous immune system and, thereby, possibly increase the number of pediatric cancer patients responding to immunotherapies that rely on antigen-specific T cell responses. For pediatric cancers that are high in stemness genes and low in MHC-peptide expression, immunotherapy approaches that are independent of MHC class I expression, such as bi-specific T cell engager antibodies (BiTE) and T cells engineered to express chimeric antigen receptors (CAR T), could be more efficient, provided that sufficient tumor specific (neo-)antigens are identified as targets [10].

## 5. Conclusions

We conclude that MHC class I and stemness assessment could serve as an important biomarker for pediatric patients with SRBCTs. MHC class I and stemness gene expression are most likely tightly connected and a result of gene expression patterns in the precursor cells these cancers originate from, which can be exploited therapeutically.

## Figures and Tables

**Table 1:** Six pediatric small round blue cell tumor types. The cohort was restricted to Affymetrix platforms (HGU133Plus2 [GPL570] and HGU133A [GPL96]). Clinical–pathological data were retrieved from the Gene Expression Omnibus (GEO) and published literature.

Dataset	Platform	Cancer Type	Clinical Data	No. Samples
GSE73038	GPL570	CNS-PNET	age, gender, OS, PFS	59
GSE34620	GPL570	ESFT	OS, EFS, age	117
GSE17679	GPL570	ESFT	OS, EFS, age, gender	32
GSE37418	GPL570	MB	age, gender, subgroup, histology	76
GSE35493	GPL570	MB	subgroup	21
GSE73038	GPL570	MB	age, gender, OS, PFS, subgroup	47
GSE16476	GPL570	NB	age, gender, PFS, OS, MYCN, INSS	88
GSE16237	GPL570	NB	age, MYC, INSS	50
GSE12460	GPL570	NB	age, gender, PFS, OS, MYCN, INSS	53
GSE13136	GPL570	NB	age, gender, OS, MYC, INSS	30
GSE66533	GPL570	RMS	fusion status (PAX3-FOXO1/PAX7-FOXO1)	58
GSE92689	GPL96	RMS	age, OS, histology	158
GSE53224	GPL570	WT	histology, gender	53
GSE31403	GPL96	WT	histology, stage, age, OS, RFS	224
GSE10320	GPL96	WT	histology, stage, age, OS, RFS	68
TOTAL		6 malignancies		1134

Abbreviations: Ewing’s sarcoma family of tumors, ESFT; medulloblastoma, MB; neuroblastoma, NB; central nervous system-primitive neuroectodermal tumor, CNS-PNET; rhabdomyosarcoma, RMS; Wilms tumor, WT; overall survival, OS; event-free survival, EFS; relapse-free survival, RFS; progression-free survival, PFS.

## Data Availability

Only publicly available datasets were analyzed in this study. Accession numbers (GSE) can be found in Table 1.

## Supplementary Materials

The following supporting information can be downloaded at: https://www.mdpi.com/article/10.3390/cancers14153584/s1, Table S1: Gene expression comparison (mean, [95% CI]) of MHC class I genes, MHC class I associated genes (antigen presenting machinery and MHC class I regulatory genes) and selected proliferation genes as indicated. Largest cohorts per cancer type are shown. Table S2: Correlation values (Spearman’s rank correlation coefficient) between mRNAsi and mean MHC class I expression within all datasets included in this study.
