# Supplementary material for: Stemness Correlates Inversely with MHC Class I Expression in Pediatric Small Round Blue Cell Tumors
Source: Cancers (Basel). 2022 Jul 22;14(15):3584. doi: 10.3390/cancers14153584 (PMC9331651; doi:10.3390/cancers14153584)
Supplement: Supplementary file 1 [file cancers-14-03584-s001.zip › cancers-1820806-supplementary.pdf]

cancers

MDPI

Supplementary Materials

Table S1. Gene expression comparison (mean, [95% CI]) of MHC class I genes, MHC class I associated genes (antigen presenting machinery and MHC class I regulatory genes) and selected proliferation genes as indicated. Largest cohorts per cancer type are shown.

GSE	Platform	Cancer Type	Gene	Gene Expression (Mean, [95% CI])
GSE73038	GPL570	CNS-PNET	B2M	12.71 [12.49–12.93]
GSE34620	GPL570	ESFT	B2M	13.02 [12.98–13.06]
GSE37418	GPL570	MB	B2M	11.20 [11.01–11.39]
GSE16476	GPL570	NB	B2M	12.77 [12.67–12.86]
GSE92689	GPL96	RMS	B2M	11.59 [11.44–11.74]
GSE31403	GPL96	WT	B2M	10.80 [10.73–10.88]
GSE73038	GPL570	CNS-PNET	HLA-A	11.52 [11.29–11.75]
GSE34620	GPL570	ESFT	HLA-A	12.41 [12.30–12.51]
GSE37418	GPL570	MB	HLA-A	11.03 [10.83–11.23]
GSE16476	GPL570	NB	HLA-A	11.32 [11.16 - 11.48]
GSE92689	GPL96	RMS	HLA-A	10.55 [10.34–10.75]
GSE31403	GPL96	WT	HLA-A	10.64 [10.54–10.73]
GSE73038	GPL570	CNS-PNET	HLA-B	10.25 [9.97–10.52]
GSE34620	GPL570	ESFT	HLA-B	10.51 [10.37–10.66]
GSE37418	GPL570	MB	HLA-B	9.15 [8.96–9.35]
GSE16476	GPL570	NB	HLA-B	11.23 [11.02–11.44]
GSE92689	GPL96	RMS	HLA-B	10.74 [10.53 - 10.94]
GSE31403	GPL96	WT	HLA-B	10.26 [10.15–10.38]
GSE73038	GPL570	CNS-PNET	HLA-C	10.31 [10.06–10.56]
GSE34620	GPL570	ESFT	HLA-C	11.07 [10.94–11.19]
GSE37418	GPL570	MB	HLA-C	9.60 [9.41–9.78]
GSE16476	GPL570	NB	HLA-C	10.71 [10.51–10.90]
GSE92689	GPL96	RMS	HLA-C	10.41 [10.23–10.59]
GSE31403	GPL96	WT	HLA-C	10.11 [10.01–10.21]
GSE73038	GPL570	CNS-PNET	HLA-E	9.03 [8.76–9.30]
GSE34620	GPL570	ESFT	HLA-E	9.52 [9.38–9.67]
GSE37418	GPL570	MB	HLA-E	7.76 [7.59–7.93]
GSE16476	GPL570	NB	HLA-E	8.75 [8.56–8.93]
GSE92689	GPL96	RMS	HLA-E	8.50 [8.39–8.62]
GSE31403	GPL96	WT	HLA-E	7.92 [7.86–7.98]
GSE73038	GPL570	CNS-PNET	HLA-F	9.21 [9.01–9.42]
GSE34620	GPL570	ESFT	HLA-F	9.23 [9.11–9.35]
GSE37418	GPL570	MB	HLA-F	8.42 [8.27–8.57]
GSE16476	GPL570	NB	HLA-F	9.20 [9.03–9.38]
GSE92689	GPL96	RMS	HLA-F	9.05 [8.93–9.18]
GSE31403	GPL96	WT	HLA-F	8.38 [8.31–8.45]
GSE73038	GPL570	CNS-PNET	HLA-G	8.02 [7.95–8.10]
GSE34620	GPL570	ESFT	HLA-G	7.04 [6.98–7.10]
GSE37418	GPL570	MB	HLA-G	7.25 [7.18–7.32]
GSE16476	GPL570	NB	HLA-G	6.75 [6.67–6.83]
GSE92689	GPL96	RMS	HLA-G	8.81 [8.76–8.86]
GSE31403	GPL96	WT	HLA-G	8.18 [8.15–8.22]
GSE73038	GPL570	CNS-PNET	IRF1	6.09 [5.94–6.23]
GSE34620	GPL570	ESFT	IRF1	6.20 [5.98–6.41]

GSE37418	GPL570	MB	IRF1	5.51 [5.42–5.59]
GSE16476	GPL570	NB	IRF1	5.62 [5.50–5.74]
GSE92689	GPL96	RMS	IRF1	6.52 [6.44–6.60]
GSE31403	GPL96	WT	IRF1	6.25 [6.21–6.29]
GSE73038	GPL570	CNS-PNET	IRF2	6.89 [6.76–7.03]
GSE34620	GPL570	ESFT	IRF2	7.05 [6.94–7.16]
GSE37418	GPL570	MB	IRF2	6.03 [5.93–6.14]
GSE16476	GPL570	NB	IRF2	5.89 [5.79–5.98]
GSE92689	GPL96	RMS	IRF2	6.19 [6.11–6.26]
GSE31403	GPL96	WT	IRF2	6.54 [6.49–6.59]
GSE73038	GPL570	CNS-PNET	NLRC5	6.45 [6.24–6.65]
GSE34620	GPL570	ESFT	NLRC5	5.83 [5.69–5.98]
GSE37418	GPL570	MB	NLRC5	6.05 [5.86–6.23]
GSE16476	GPL570	NB	NLRC5	6.31 [6.10–6.51]
GSE92689	GPL96	RMS	NLRC5	NA
GSE31403	GPL96	WT	NLRC5	NA
GSE73038	GPL570	CNS-PNET	NFKB1	7.37 [7.23–7.52]
GSE34620	GPL570	ESFT	NFKB1	6.70 [6.58–6.81]
GSE37418	GPL570	MB	NFKB1	7.13 [7.03–7.24]
GSE16476	GPL570	NB	NFKB1	7.27 [7.17–7.37]
GSE92689	GPL96	RMS	NFKB1	8.09 [8.04–8.14]
GSE31403	GPL96	WT	NFKB1	7.48 [7.45–7.52]
GSE73038	GPL570	CNS-PNET	NFKB2	5.01 [4.91–5.11]
GSE34620	GPL570	ESFT	NFKB2	5.41 [5.33–5.49]
GSE37418	GPL570	MB	NFKB2	4.32 [4.24–4.39]
GSE16476	GPL570	NB	NFKB2	4.63 [4.56–4.70]
GSE92689	GPL96	RMS	NFKB2	4.90 [4.86–4.92]
GSE31403	GPL96	WT	NFKB2	5.39 [5.37–5.41]
GSE73038	GPL570	CNS-PNET	TAP1	7.99 [7.74–8.25]
GSE34620	GPL570	ESFT	TAP1	7.74 [7.64–7.83]
GSE37418	GPL570	MB	TAP1	6.94 [6.76–7.13]
GSE16476	GPL570	NB	TAP1	6.85 [6.68–7.03]
GSE92689	GPL96	RMS	TAP1	7.24 [7.15–7.33]
GSE31403	GPL96	WT	TAP1	6.69 [6.63–6.74]
GSE73038	GPL570	CNS-PNET	TAP2	5.70 [5.60–5.79]
GSE34620	GPL570	ESFT	TAP2	5.32 [5.27–5.36]
GSE37418	GPL570	MB	TAP2	4.70 [4.62–4.79]
GSE16476	GPL570	NB	TAP2	4.29 [4.22–4.35]
GSE92689	GPL96	RMS	TAP2	6.08 [6.04–6.11]
GSE31403	GPL96	WT	TAP2	5.36 [5.34–5.39]
GSE73038	GPL570	CNS-PNET	TAPBP	6.16 [6.04–6.28]
GSE34620	GPL570	ESFT	TAPBP	6.03 [5.96–6.10]
GSE37418	GPL570	MB	TAPBP	5.53 [5.46–5.60]
GSE16476	GPL570	NB	TAPBP	5.28 [5.20–5.35]
GSE92689	GPL96	RMS	TAPBP	6.37 [6.32–6.41]
GSE31403	GPL96	WT	TAPBP	6.82 [6.78–6.86]
GSE73038	GPL570	CNS-PNET	MKI67	6.87 [6.66–7.08]
GSE34620	GPL570	ESFT	MKI67	6.38 [6.25–6.50]
GSE37418	GPL570	MB	MKI67	6.94 [6.77–7.12]
GSE16476	GPL570	NB	MKI67	6.38 [6.18–6.58]
GSE92689	GPL96	RMS	MKI67	7.37 [7.29–7.45]
GSE31403	GPL96	WT	MKI67	7.44 [7.39–7.49]
GSE73038	GPL570	CNS-PNET	BUB1B	8.69 [8.41–8.97]

GSE34620	GPL570	ESFT	BUB1B	8.15 [7.98–8.32]
GSE37418	GPL570	MB	BUB1B	8.00 [7.77–8.23]
GSE16476	GPL570	NB	BUB1B	7.88 [7.62–8.13]
GSE92689	GPL96	RMS	BUB1B	7.78 [7.64–7.92]
GSE31403	GPL96	WT	BUB1B	8.61 [8.54–8.68]
GSE73038	GPL570	CNS-PNET	CCNB1	8.75 [8.49–9.00]
GSE34620	GPL570	ESFT	CCNB1	7.63 [7.47–7.79]
GSE37418	GPL570	MB	CCNB1	7.83 [7.62–8.04]
GSE16476	GPL570	NB	CCNB1	7.21 [6.97–7.46]
GSE92689	GPL96	RMS	CCNB1	7.65 [7.49–7.81]
GSE31403	GPL96	WT	CCNB1	7.88 [7.80–7.97]

Table S2. Correlation values (Spearman's rank correlation coefficient) between mRNAsi and mean MHC class I expression within all datasets included in this study.

GSE	Platform	Cancer Type	n	Subtype	r	*p* Adjust
GSE73038	GPL570	CNS-PNET	59	NA	−0.49	<0.001
GSE34620	GPL570	ESFT	117	NA	−0.46	<0.001
GSE17679	GPL570	ESFT	32	NA	−0.63	<0.001
GSE37418	GPL570	MB	76	NA	−0.24	0.064
GSE35493	GPL570	MB	21	NA	−0.18	0.592
GSE73038	GPL570	MB	47	NA	−0.39	0.014
GSE16476	GPL570	NB	88	NA	−0.41	<0.001
	GPL570	NB	16	MYCN amplified	−0.63	0.009
	GPL570	NB	72	MYCN non amplified	−0.39	0.001
GSE16237	GPL570	NB	50	NA	−0.54	<0.001
GSE12460	GPL570	NB	53	NA	−0.63	<0.001
	GPL570	NB	14	MYCN amplified	−0.61	0.021
	GPL570	NB	32	MYCN non amplified	−0.52	0.005
GSE13136	GPL570	NB	30	NA	−0.54	0.005
	GPL570	NB	10	MYCN amplified	−0.71	0.025
	GPL570	NB	20	MYCN non amplified	−0-5	0.025
GSE66533	GPL570	RMS	58	NA	−0.54	<0.001
GSE92689	GPL96	RMS	158	NA	−0.5	<0.001
	GPL96	RMS	65	embryonal	−0.61	<0.001
	GPL96	RMS	65	alveolar	−0.45	<0.001
	GPL96	RMS	23	other	−0.47	0.252
GSE53224	GPL570	WT	53	NA	−0.84	<0.001
	GPL570	WT	34	blastemal	−0.84	<0.001
	GPL570	WT	1	epithelial	NA	NA
	GPL570	WT	1	stromal	NA	NA
	GPL570	WT	6	triphasic	−0.66	0.156
GSE31403	GPL96	WT	224	NA	−0.39	<0.001
	GPL96	WT	81	blastemal	−0.4	<0.001
	GPL96	WT	19	epithelial	−0.81	<0.001
	GPL96	WT	5	stromal	−0.3	0.624
	GPL96	WT	115	triphasic	−0.29	0.002
GSE10320	GPL96	WT	68	NA	−0.44	<0.001
	GPL96	WT	29	blastemal	−0.39	0.037
	GPL96	WT	5	epithelial	−0.9	0.037
	GPL96	WT	2	stromal	NA	NA
	GPL96	WT	29	triphasic	−0.42	0.037
